# A starvation-induced regulator, RovM, acts as a switch for planktonic/biofilm state transition in *Yersinia pseudotuberculosis*

**DOI:** 10.1038/s41598-017-00534-9

**Published:** 2017-04-04

**Authors:** Ruoxi Zhao, Yunhong Song, Qingyun Dai, Yiwen Kang, Junfeng Pan, Lingfang Zhu, Lei Zhang, Yao Wang, Xihui Shen

**Affiliations:** State Key Laboratory of Crop Stress Biology for Arid Areas and College of Life Sciences, Northwest A&F University, Yangling, Shaanxi 712100 China

## Abstract

The transition between the planktonic state and the biofilm-associated state is a key developmental decision for pathogenic bacteria. Biofilm formation by *Yersinia pestis* is regulated by *hmsHFRS* genes (β-1, 6-N-acetyl-D-glucosamine synthesis operon) in its flea vector and *in vitro*. However, the mechanism of biofilm formation in *Yersinia pseudotuberculosis* remains elusive. In this study, we demonstrate that the LysR-type regulator RovM inversely regulates biofilm formation and motility in *Y. pseudotuberculosis* by acting as a transcriptional regulator of these two functions. RovM is strongly induced during growth in minimal media but strongly repressed in complex media. On one hand, RovM enhances bacterial motility by activating the expression of FlhDC, the master regulator of flagellar genes, via the recognition of an operator upstream of the *flhDC* promoter. On the other hand, RovM represses β-GlcNAc production under nutrition-limited conditions, negatively regulating *hmsHFRS* expression by directly binding to the −35 element of its promoter. Compared to wild-type bacteria, the *rovM* mutant established denser biofilms and caused more extensive mortality in mice and silkworm larvae. These results indicate that RovM acts as a molecular switch to coordinate the expression of genes involved in biofilm formation and motility in response to the availability of nutrients.

## Introduction

During their life cycles, bacterial pathogens must often transition rapidly between planktonic and sessile states to adapt to changing environmental conditions^1–4^. Sessile bacteria may form biofilms for protection from diverse environmental stressors, including conventional antimicrobial agents and immune reactions. These biofilms have significant impacts on bacterial virulence in chronic infections^5, 6^. Understanding the molecular mechanism underlying biofilm regulation in pathogens is therefore essential to the development of innovative treatment strategies. The process of biofilm formation in response to environmental signals is dynamic and complex. Molecular switches including sigma factors, transcription factors, small regulatory RNAs, and secondary messengers control the transition between motile and biofilm-associated states by modulating mutually exclusive motility and adhesion-related biofilm matrix components such as amyloid curli fimbriae, cellulose, and β-(1 → 6)-poly-N-acetyl-D-glucosamine (β-GlcNAc)^4, 7–10^.

Among a remarkable range of pathogens, the transition from the planktonic state to a biofilm is mediated by one central switch, (3′–5′)-cyclic-diguanosine monophosphate (c-di-GMP), which reduces motility and promotes biofilm formation at high concentrations^11–14^. Additionally, CsgD is reported as an important switch, as it induces expression of the *csgBAC* operon, required for the production of curli fimbriae and cellulose, as well as the production of c-di-GMP^15^. Most regulators of CsgD and c-di-GMP concentration have also been identified as planktonic/biofilm switches, including McaS^16^, RpoS^17–19^, and Hha^20^. On the other hand, switches that activate dispersal of planktonic cells from a biofilm are also essential, as they permit bacteria to escape the confines of the biofilm and colonize new locations^5^. Unlike other molecular switches, CsrA, a small RNA-binding protein, activates biofilm dispersal by inhibiting the synthesis of β-GlcNAc via direct repression of *pgaA* translation^21^, while also enhancing motility by protecting the transcript of the flagellar master regulator FlhDC from degradation^22^. Cooperation among multiple distinct switches enables pathogens to withstand harsh environmental conditions and encourages successful infection by allowing rapid changes between the motile and biofilm states.

*Yersinia pseudotuberculosis* is a Gram-negative food-borne enteric pathogen that causes a variety of intestinal and extraintestinal infections. In *Y. pseudotuberculosis*, swimming motility is primarily controlled by the expression of the flagellar master regulator *flhDC*. Previously studies have shown that the YpsRI and YtbRI quorum sensing systems^23^, CsrA^24^, OmpR^25^, and RpoS^26^ indirectly modulate swimming motility by controlling the expression of *flhDC*. The *Y. pseudotuberculosis hmsHFRS* operon is responsible for synthesis and transport of the exopolysaccharide β-GlcNAc, the primary dry component of the biofilm matrix^27^. It had been observed that the HmsHFRS system is subject to post-transcriptional regulation in response to the c-di-GMP messenger in *Yersinia pestis* ^28^. HmsT and HmsD are the only two diguanylate cyclases that catalyze c-di-GMP synthesis. RcsAB is also a major repressor of *Yersinia* biofilm development via influencing *hmsCDE*, *hmsT*, and *hmsHFRS* expression^29–31^. Despite these previous studies, we lack significant information regarding control of the motile/biofilm state transition in *Y. pseudotuberculosis*, as well as the underlying molecular mechanisms of this transition.

A LysR-type transcription factor in *Y. pseudotuberculosis*, RovM, was known to attenuate *Yersinia* virulence by repressing the expression of the global virulence regulator RovA, and also has been shown to control flagellar motility through a currently unknown mechanism^32^. RovM acts as both an activator and a repressor fine-tuning expression of the type VI secretion system T6SS4- and AR3-dependent acid survival systems in *Y. pseudotuberculosis* in response to the availability of nutrients^33^. Although RovM homologs in other bacteria are known to regulate various cellular processes including biofilm formation^34, 35^, it remains unknown whether RovM controls biofilm gene expression in *Y. pseudotuberculosis*. In this study, we provide evidence that RovM, acting as a motile-sessile state switch, regulates motility and biofilm formation based on nutrient availability.

## Results

### RovM enhances motility via transcriptional activation of *flhDC* expression

**Figure 1:**
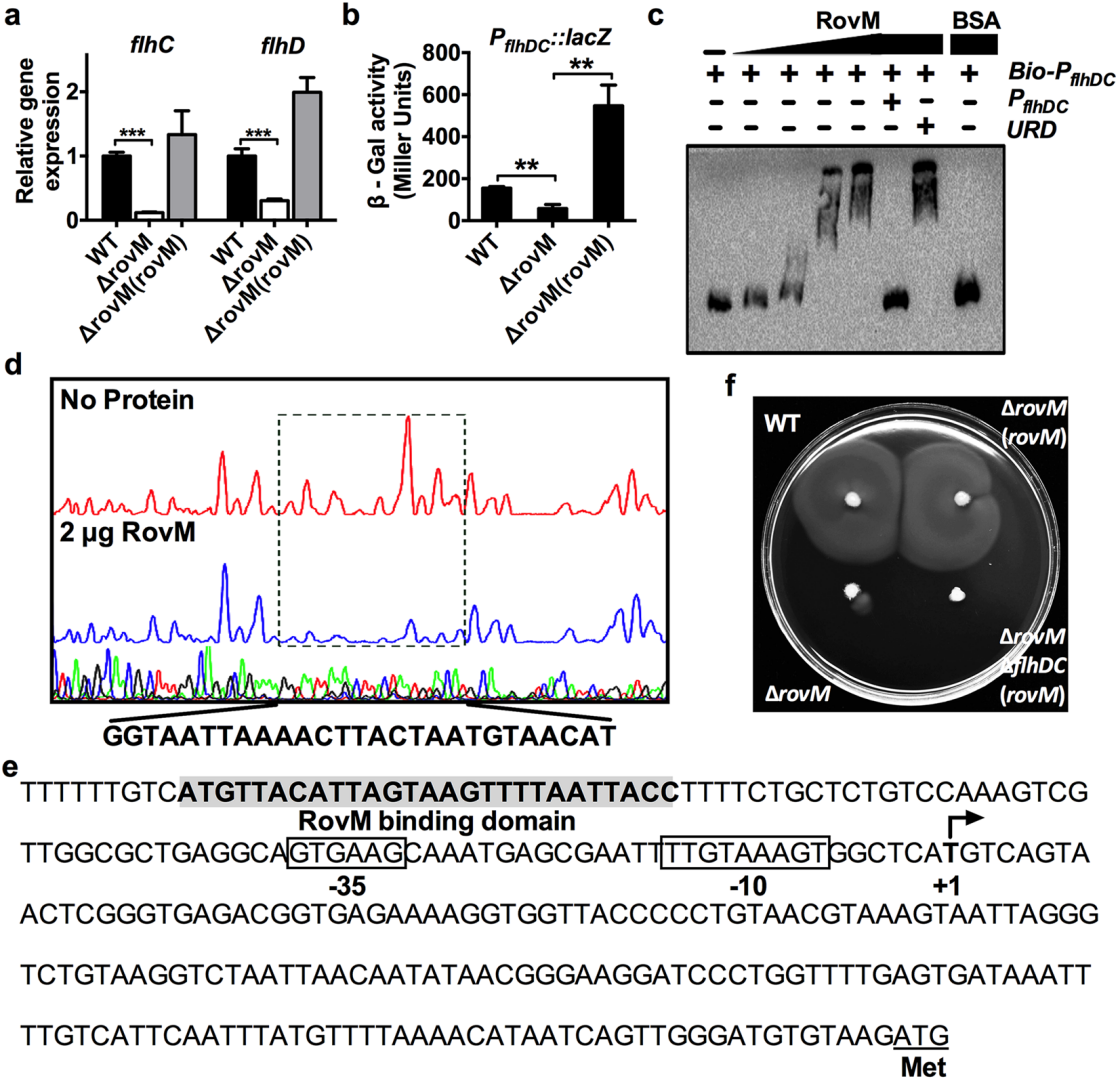
RovM controls bacteria motility by directly regulating *flhDC* expression. (**a**,**b**) RovM enhances the expression of the *flhDC* operon. The relative expression measured by quantitative RT-PCR (**a**) or the β-galactosidase activity (**b**) in the indicated bacterial strains was determined. (**c**) RovM binds the *flhDC* promoter. Biotin-labelled probe, unlabelled probe or an unrelated fragment was incubated with RovM [0, 0.13, 0.27, 0.54 and 0.108 µM] or BSA [5 µM]. The protein-DNA complexes were detected by streptavidin-conjugated HRP and chemiluminescent substrate. Unlabelled promoter was added to determine the binding specificity of RovM. Bio-P_*flhDC*_: biotin-labelled *flhDC* promoter; P_*flhDC*_: unlabelled *flhDC* promoter; URD: unrelated fragment (uncropped version was shown in Fig. S4a). (**d**) Identification of the RovM-binding site within the *flhDC* promoter using a DNase I footprinting assay. (**e**) Nucleotide sequence of the *flhDC* promoter region. Putative −35 and −10 elements of the *flhDC* promoter are boxed. +1 denotes the transcription start point. The RovM-binding site identified with the DNase I footprinting assay was indicated by shading. (**f**) Motility of the *Y. pseudotuberculosis* YPIII, Δ*rovM* mutant, Δ*rovM*(*rovM*) and Δ*rovM*Δ*flhDC*(*rovM*) strains on semi-solid plates. Data shown are the average of three independent experiments; error bars indicate SD from three independent experiments. **P < 0.01; ***P < 0.001.

Previously, RovM (*ypk_1559*) was reported to enhance bacterial motility and flagellar synthesis in *Y. pseudotuberculosis* ^24, 32^, however, the underlying mechanism has not been identified. Since FlhDC is the master regulator of flagellar production, we sought to determine whether RovM enhances bacterial motility by altering the expression of the flagellar master regulator *flhDC* (*ypk_1745-1746*) in *Y. pseudotuberculosis*. To this end, we quantified the expression of *flhD* and *flhC* in the wild-type, the Δ*rovM* mutant, and the complemented strain during the late exponential phase. As shown in Fig. 1a, the expression of *flhD* and *flhC* in the Δ*rovM* mutant was significantly lower than in the wild-type and complemented strains. Similarly, expression of the *P*_*flhDC*_ *::lacZ* transcriptional fusion reporter, which monitors the expression of *flhDC*, was considerably lower in the Δ*rovM* mutant compared with the wild-type strain, whereas expression of the *rovM* gene in Δ*rovM* resulted in a marked increase in the expression of the *P*_*flhDC*_ *::lacZ* reporter (Fig. 1b).

To determine whether RovM directly regulates *flhDC* expression, we examined the interaction between RovM and the *flhDC* promoter using an electrophoretic mobility shift assay (EMSA). Incubation of a probe harboring the *flhDC* promoter sequence [−1 to −652] relative to the ATG start codon of the first open reading frame (ORF) of the *flhDC* operon with purified His_6_-RovM led to the formation of protein-DNA complexes, and the abundance of such complexes depended on the amount of RovM present (Fig. 1c). The interactions between His_6_-RovM and the *flhDC* promoter are specific because excessive unlabeled probe abolished the formation of the protein-DNA complex. However, an unrelated fragment amplified from the coding region of the *flhDC* operon could not disrupt the formation of such complexes (Fig. 1c). Next, we identified a protected region of DNA with high affinity to RovM extending from −242 to −268 bp upstream of the start codon of the *flhD* ORF using DNase I footprinting analysis (Fig. 1d,e). Our data demonstrate that RovM plays a crucial role in flagellar synthesis and motility by directly regulating the expression of *flhDC*. This conclusion was further supported by the finding that complementation of *rovM* eliminated the motility defects of the Δ*rovM* mutant but failed to restore motility of the Δ*rovM*Δ*flhDC* double mutant (Fig. 1f).

### RovM represses β-GlcNAc production under nutrition-limited conditions

**Figure 2:**
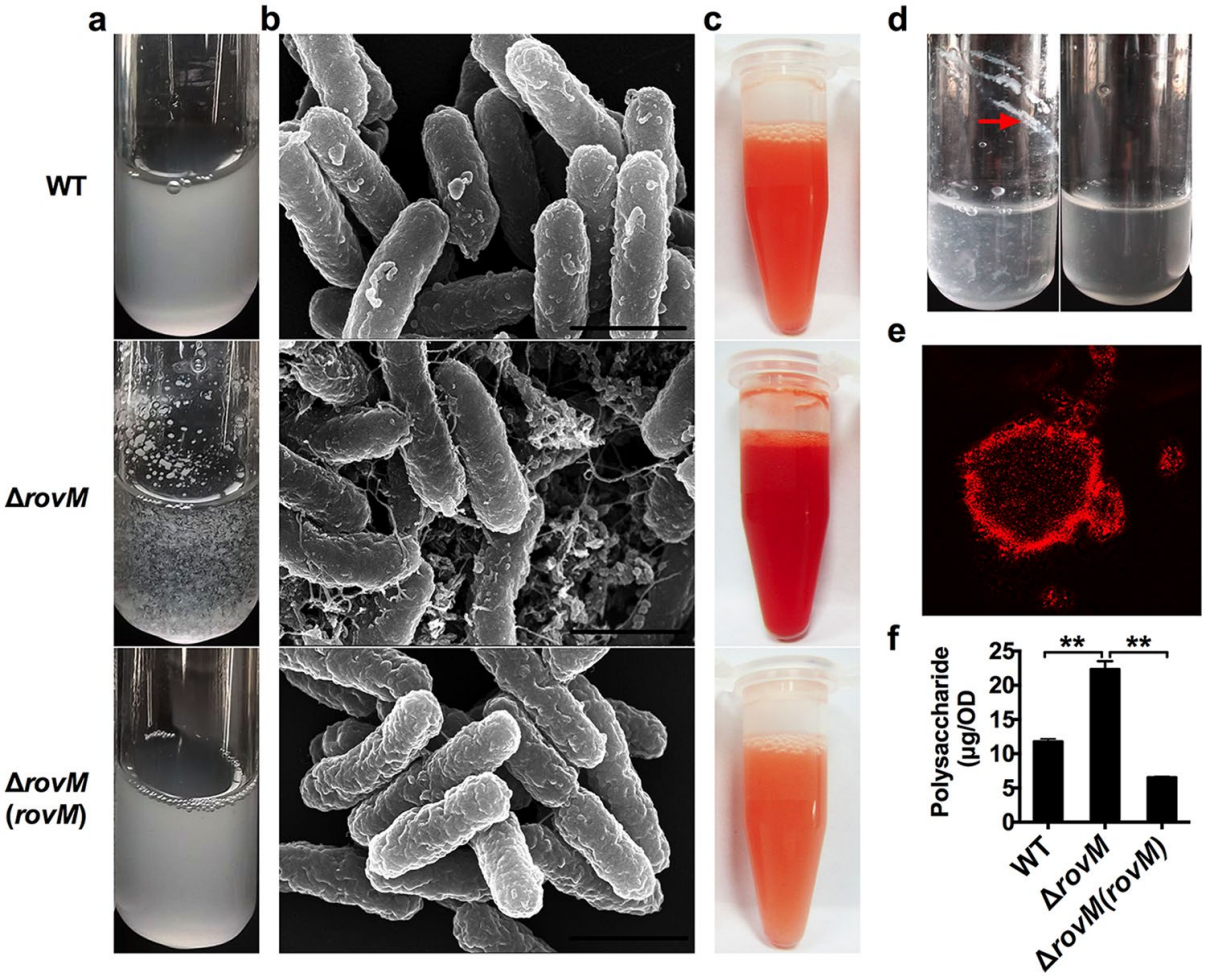
Deletion of *rovM* causes aggregates. (**a,b**) Observation of cell-cell aggregates formed by wild-type strain, Δ*rovM* and Δ*rovM*(*rovM*) grown in M9 medium in tubes (**a**) and under SEM (**b**). Scale bar = 10 μm. (**c**) WT, Δ*rovM* and Δ*rovM*(*rovM*) grown to late exponential phase in M9 medium stained by Congo red. (**d**) Cell aggregates formed in Δ*rovM* culture were treated with (Right) or without (Left) sodium metaperiodate (1 M) at 4 °C for 24 h. Red arrow indicates aggregate adhered on tube. (**e**) Fluorescence microscopy image of Δ*rovM* aggregates stained by WGA-R. (**f**) Extracellular polysaccharide content in the *Y. pseudotuberculosis* WT, Δ*rovM* and Δ*rovM*(*rovM*) determined by the MBTH assay. Data shown are the average of three independent experiments; error bars indicate SD from three independent experiments. **P < 0.01.

We observed that liquid suspensions of the Δ*rovM* mutant grown in nutrition-limited M9 medium, but not those grown in nutrient-rich YLB medium, formed large aggregates that settled quickly when left standing (Fig. 2a). However, the wild-type strain formed fewer aggregates, which did not settle out of suspension when grown under the same conditions. Moreover, the formation of aggregates in the Δ*rovM* mutant could be rescued by providing the *rovM* gene in *trans* (Fig. 2a). To investigate this phenomenon in more detail, we used scanning electron microscopy (SEM) to visualize aggregates in late exponential phase cultures of the wild-type strain, Δ*rovM* mutant, and the complemented strain. As shown in Fig. 2b, the Δ*rovM* mutant formed large aggregates in which bacteria appear to be embedded within a web-like matrix. However, this matrix was not observed in the wild-type or complemented strains (Fig. 2b).

As polysaccharides are known to be a major component of bacterial aggregates, we reasoned that the Δ*rovM* mutant formed large bacterial aggregates due to over-production of polysaccharides. To test this hypothesis, we monitored the presence of polysaccharides in aggregates of the wild-type, Δ*rovM* mutant, and complemented strains using a Congo red (CR) staining assay. As predicted, the Δ*rovM* mutant exhibited a stronger CR-positive phenotype than the wild-type and complemented strains (Fig. 2c). Furthermore, treatment of the Δ*rovM* aggregates with metaperiodate, a chemical known to degrade polysaccharides by oxidizing the carbon atoms (3 and 4) bearing vicinal hydroxyl groups and cleaving their C-C bonds^36^, resulted in near-complete disruption of the aggregates (Fig. 2d). These results suggest that RovM might be involved in the repression of polysaccharide production. Consistent with the report that β-GlcNAc is the major polysaccharide in the extracellular matrix of *Y. pseudotuberculosis* ^27^, the Δ*rovM* aggregates stained positively using a wheat germ agglutinin-rhodamine (WGA-R) conjugate (Fig. 2e), which is known to bind specifically to β-GlcNAc and its oligomers. To more quantitatively assess the production of polysaccharides, we determined the total amount of β-GlcNAc produced by each strain using a 3-methyl-2-benzothiazoninone hydrazone (MBTH) assay^37^. The Δ*rovM* mutant produced twice as much β-GlcNAc as the wild-type strain, while the complemented strain produced the least β-GlcNAc (Fig. 2f). These results suggest that RovM suppresses β-GlcNAc production in *Y. pseudotuberculosis* through regulating the *hms* operon.

### RovM directly represses *hmsHFRS* expression

**Figure 3:**
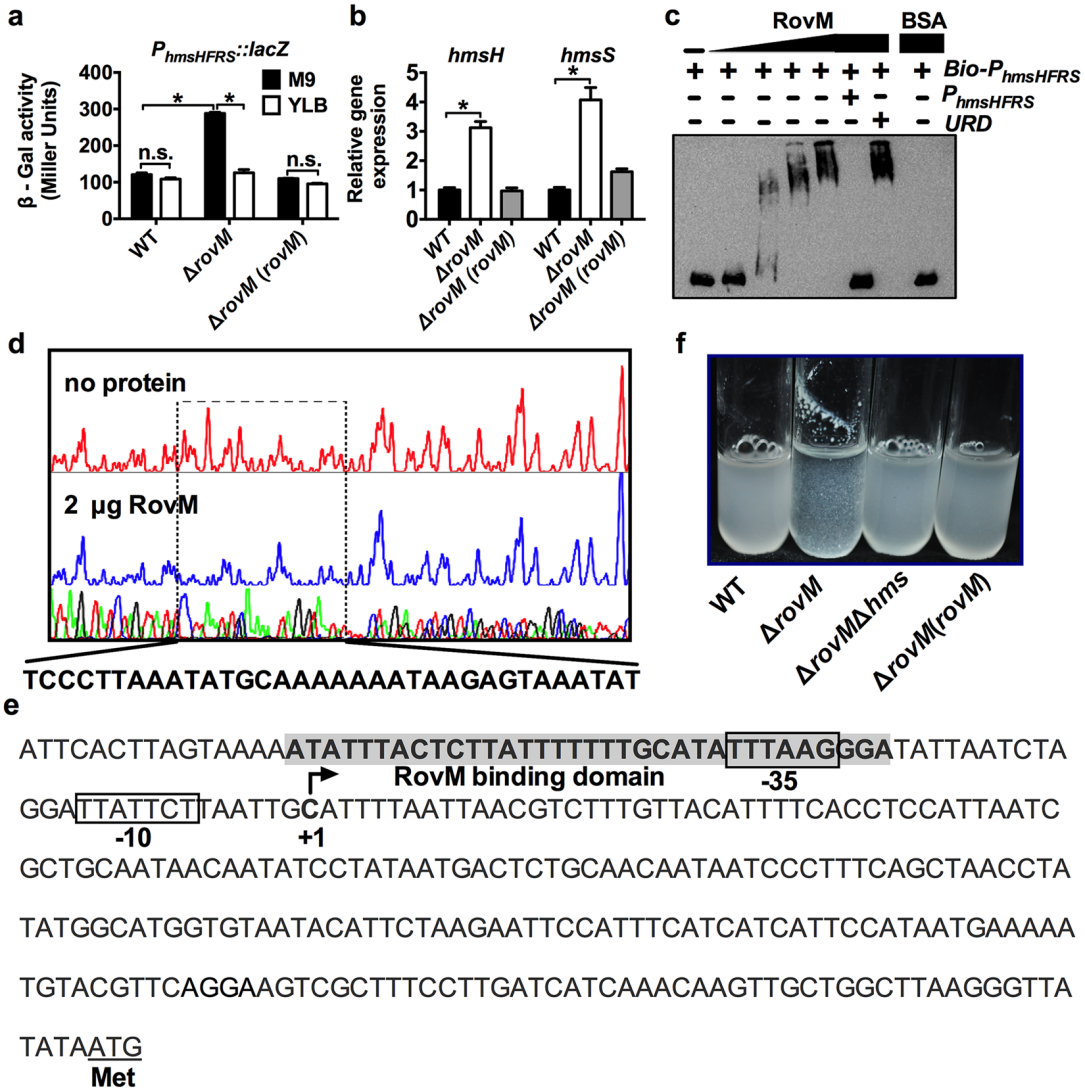
RovM represses *hmsHMSF* expression directly. (**a,b**) RovM repress the expression of the *hms* operon. The β-galactosidase activity (**a**) or relative expression measured by quantitative RT-PCR (**b**) in the indicated bacterial strains was determined. (**c**) RovM binds the *hmsHFRS* promoter. Biotin-labelled probe, unlabelled probe or an unrelated fragment was incubated with RovM [0, 0.13, 0.27, 0.54 and 0.108 µM] or BSA [5 µM]. The protein-DNA complexes were detected by streptavidin- conjugated HRP and chemiluminescent substrate. Unlabelled promoter was added to determine the binding specificity of RovM. Bio-P_*hmsHFRS*_: biotin-labelled *hmsHFRS* promoter; P_*hmsHFRS*_: unlabelled *hmsHFRS* promoter; URD: unrelated fragment (uncropped version was shown in Fig. S4b). (**d**) Identification of the RovM-binding site within the *hmsHFRS* promoter using a DNase I footprinting assay. (**e**) Nucleotide sequences of the *hmsHFRS* promoter region. Putative −35 and −10 elements of the *hmsHFRS* promoter are boxed. +1 denotes the transcription start point. The RovM-binding sites identified using the DNase I footprinting assays are indicated by shading. (**f**) Aggregates formed in WT, Δ*rovM*, Δ*rovM*Δ*hmsHFR* and Δ*rovM*(*rovM*) grown in M9 to the late exponential phase. Data are presented as the mean values ± SD calculated from three sets of independent experiments. *P < 0.05; n.s., not significant.

To verify the role of RovM in the repression of β-GlcNAc production, we investigated the effect of RovM on the expression of *hmsHFRS* (*ypk_2241-2238*), the gene locus known to be responsible for synthesis and translocation of β-GlcNAc in *Y. pseudotuberculosis*. To this end, we introduced a single copy of the *P*_*hmsHFRS*_::*lacZ* transcriptional reporter fusion into the chromosome of the *Y. pseudotuberculosis* wild-type strain, the Δ*rovM* mutant, and the complemented strain. We then quantitatively assessed the LacZ activity of the resulting strains. As shown in Fig. 3a, *P*_*hmsHFRS*_::*lacZ* promoter activity increased dramatically in the Δ*rovM* mutant grown in nutrition-limited M9 medium, and this increase was absent in the complemented strain. However, increased *P*_*hmsHFRS*_::*lacZ* promoter activity was not observed in the Δ*rovM* mutant grown in nutrient-rich YLB medium. We also confirmed the negative regulation of *hmsHFRS* by RovM in M9 medium through qRT-PCR analysis, which revealed that expression of the *hmsH* and *hmsR* genes was enhanced approximately 4- to 8-fold in the Δ*rovM* mutant relative to the wild-type strain and the complemented strain (Fig. 3b). These data suggest that RovM represses β-GlcNAc production by negatively regulating *hmsHFRS* expression under nutrient-limited conditions.

To further investigate whether expression of *hmsHFRS* is regulated directly by RovM, we performed an EMSA assay. Incubation of His_6_-RovM with a 361-bp *hmsHFRS* promoter inhibited the mobility of the probe (Fig. 3c), which indicates direct binding of this protein to the *hmsHFRS* promoter. Furthermore, the amount of the protein-DNA complexes increased in response to increased levels of His_6_-RovM. The interactions between His_6_-RovM and the *hmsHFRS* promoter are specific since excessive unlabeled probe abolished the formation of the protein-DNA complex; similarly, an unrelated fragment amplified from the coding region of the *hmsHFRS* operon could not disrupt the formation of such complexes (Fig. 3c). DNase I footprinting analysis revealed a region protected from DNase I digestion extending from −245 to −279 bp upstream of the start codon of the *hmsH* gene (Fig. 3d). This region overlapped partially with the putative conserved −35 element identified by the program for prediction of bacterial promoters, BPROM (Fig. 3e). Collectively, these results indicate that RovM represses *hmsHFRS* expression by binding directly to its promoter. Consistent with these conclusions, deletion of *rovM* from the wild-type strain resulted in the formation of bacterial aggregates in M9 medium due to the over-production of β-GlcNAc. However, deletion of *rovM* from the Δ*hmsHFR* mutant caused bacterial aggregates not to form under the same conditions (Fig. 3f), further confirming the role of RovM in inducing bacterial auto-aggregation by directly repressing production of β-GlcNAc, encoded by *hmsHFRS*.

### RovM inhibits biofilm formation by suppressing β-GlcNAc production

**Figure 4:**
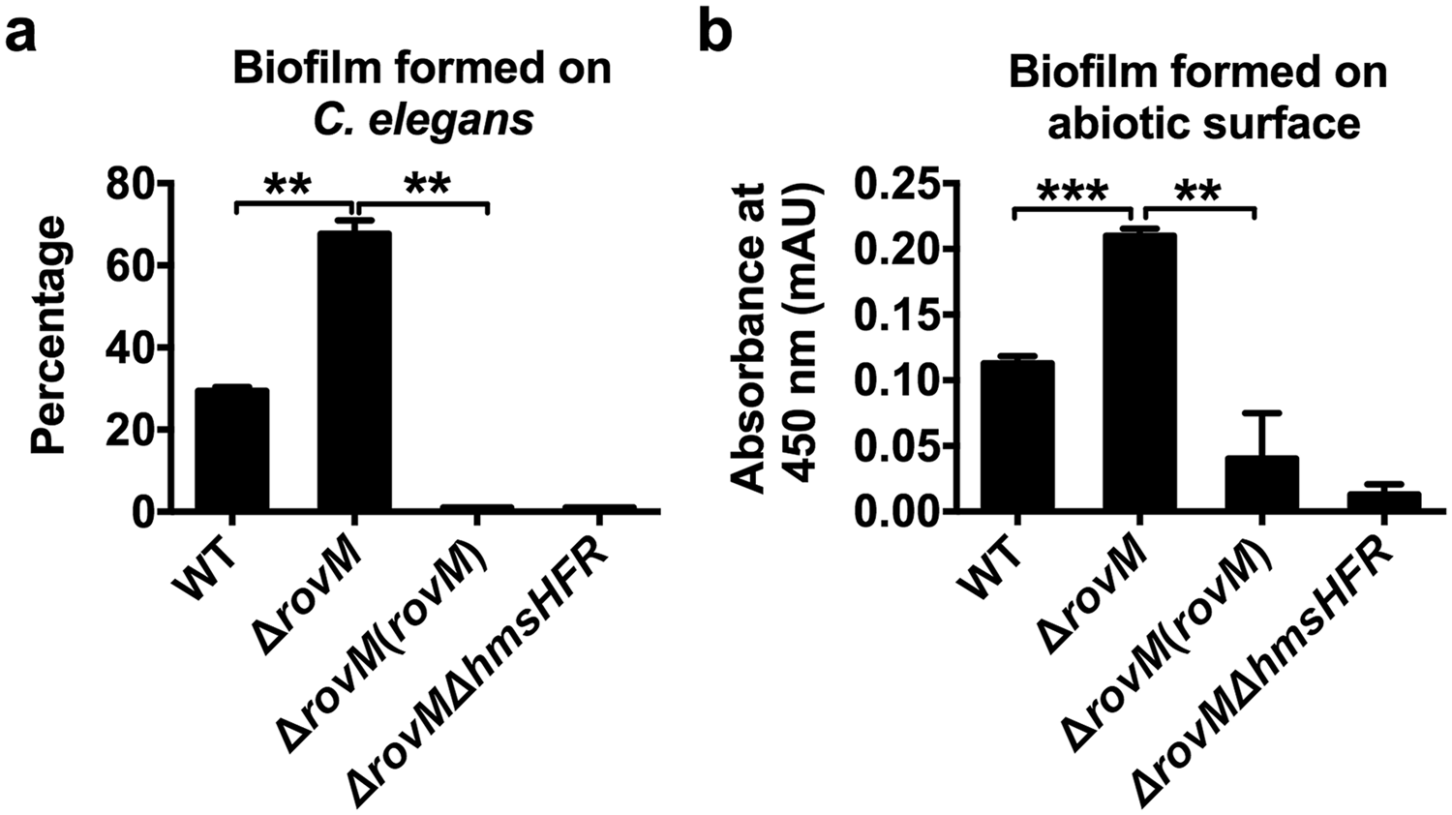
RovM represses biofilm formation. (**a**) Biofilm severity as a measurement of biofilm formation on *C. elegans* by wild-type strain, Δ*rovM*, Δ*rovM*(*rovM*) and Δ*rovM*Δ*hmsHFR*. (**b**) Biofilm formed on abiotic surface (in 96-well plates) by the indicated strains. Data shown are the average of three independent experiments; error bars indicate SD from three independent experiments. *P < 0.05; **P < 0.01; ***P < 0.001.

Since the *Y. pseudotuberculosis* biofilm matrix is primarily composed of β-GlcNAc exopolysaccharide and the *hmsHFRS* gene locus is essential for biofilm formation in *Y. pseudotuberculosis* ^27^, we hypothesized that RovM plays a role in *Y. pseudotuberculosis* biofilm formation. To test this hypothesis, we used the nematode *Caenorhabditis elegans* as a biotic surface on which to study biofilm formation by *Y. pseudotuberculosis*. Biofilm assays were performed using the *Y. pseudotuberculosis* wild-type, the Δ*rovM* mutant and the complemented strain labeled with the constitutive GFP-plasmid pKEN-GFP mutant3*. Biofilm severity indices were calculated 24 h post-infection. Each nematode was assigned a score between 0 and 3, with 0 representing the lowest level of biofilm formation and 3 representing the highest level of biofilm formation (Fig. S1a–d)^38^. These results revealed that Δ*rovM* formed more vigorous biofilms than the wild-type strain in this *C. elegans* model. Strikingly, there was no biofilm formation observed on nematodes infected with the complemented strain (Fig. 4a). This result was further confirmed by examining biofilm formation on abiotic surfaces (Fig. 4b). Consistent with the report that the *hmsHFRS* gene locus is essential for biofilm formation in *Y. pseudotuberculosis*, there was no biofilm formation observed on the nematodes infected with the Δ*rovM*Δ*hmsHFR* double mutant (Fig. 4). Together, these findings indicate that RovM plays a negative role in biofilm formation via suppressing the production of β-GlcNAc exopolysaccharide.

### RovM attenuates bacterial virulence by repressing *hms* expression

**Figure 5:**
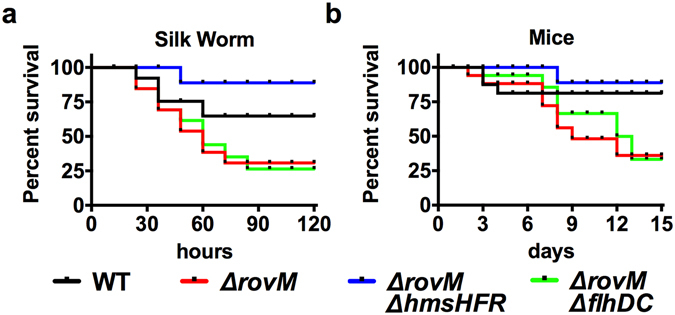
RovM attenuates bacterial virulence by repressing *hms* expression. (**a**) Bacterial strains grown in M9 were washed twice in sterilized PBS and injected into larval silkworm. 5 × 10^8^ bacteria were applied to different groups of larvae (n = 25/strain), and the survival rate was monitored every 12 h for 6 days. (**b**) The same bacteria were used for orogastric infection of 6–8 weeks old female C57BL/6 mice. For survival assays 3 × 10^9^ bacteria of each strain were applied to different groups of mice (n = 10/strain), and the survival rate of the mice was determined by monitoring the survival daily for 3 weeks. Similar results were obtained in three independent experiments, and data shown are from one representative experiment done in triplicate.

RovM was previously reported to attenuate virulence by repressing the expression of *rovA*, which controls expression of virulence genes in *Y. pseudotuberculosis* ^32^. Since biofilm formation is crucial for bacterial virulence, we hypothesized that the effect of RovM on *Y. pseudotuberculosis* virulence was also mediated by regulation of *hms*. We thus tested bacterial virulence by injecting larval silkworms with the wild-type strain, Δ*rovM*, Δ*rovM*Δ*hms*, and Δ*rovM*Δ*flhDC*. The Δ*rovM* mutant caused more than 60% mortality within 72 h of inoculation. Larvae infected with mutants lacking *rovM* and *flhDC* survived at a similar rate, while the wild-type bacteria were less virulent. Notably, mutations in both *hms* and *rovM* caused near-complete loss of the virulence to larvae (Fig. 5a), implying that regulation of *hms* is essential to the virulence of Δ*rovM*. This result was confirmed by orogastrically inoculating relevant bacterial strains into C57BL/6 mice (Fig. 5b). Together, these results indicate that in addition to repressing the expression of *rovA*, RovM attenuates bacterial virulence by repressing *hms* expression and biofilm formation.

### RovM coordinates the planktonic/biofilm state transition

**Figure 6:**
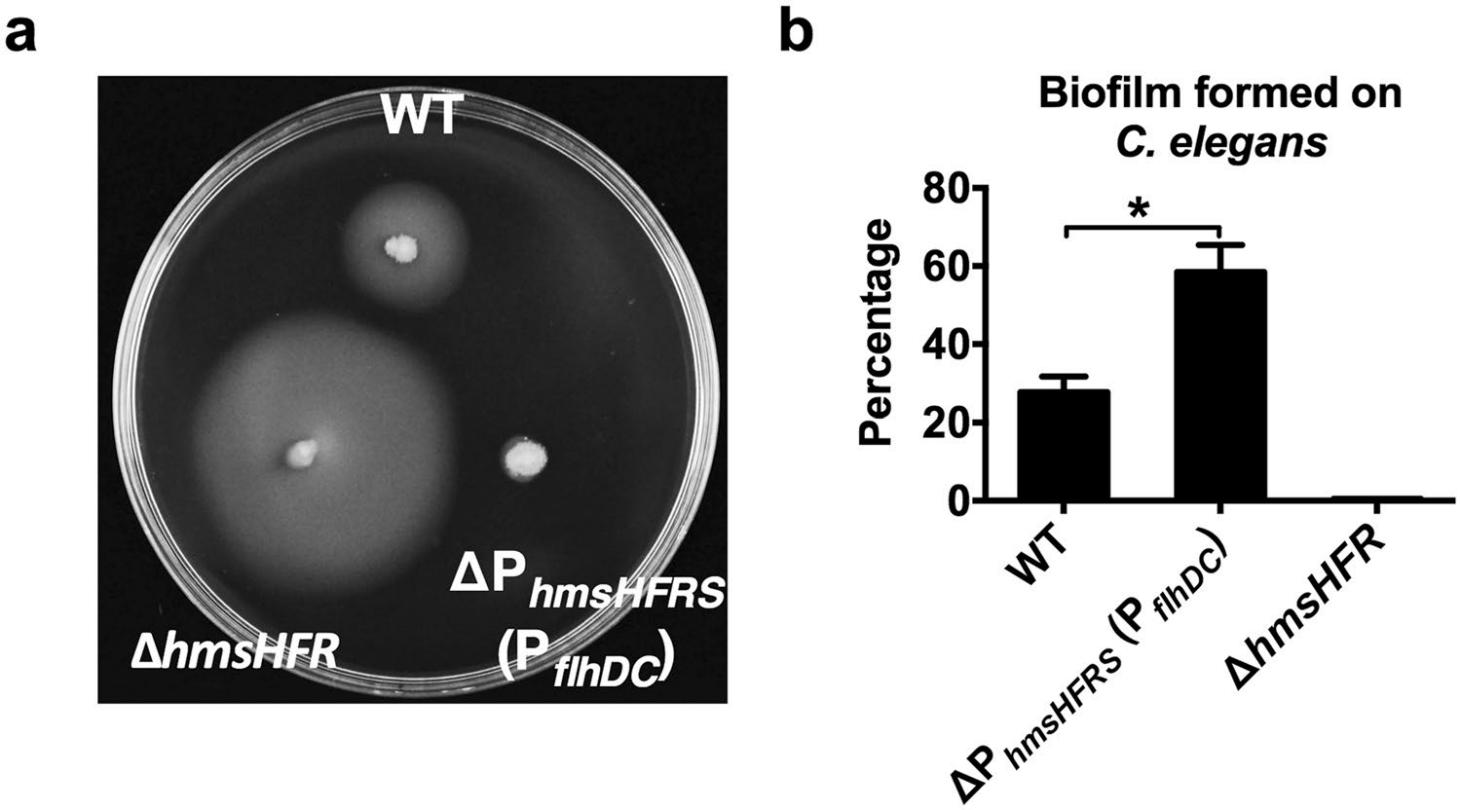
RovM modulates biofilm/motility transition through reversely regulating the expression of *flhDC* and *hmsHFRS*. (**a**) Mobility of WT, Δ*hmsHFR* and the promoter replacement strain Δ*P*_*hmsHFRS*_(*P*_*flhDC*_) on semi-solid agar plates. (**b**) Biofilm formed on *C. elegans* by WT, Δ*P*_*hmsHFRS*_(*P*_*flhDC*_) and Δ*hmsHFR*. Data shown are the average of three independent experiments; error bars indicate SD from three independent experiments. *P < 0.05; **P < 0.01; ***P < 0.001.

Because motility and adhesion are mutually exclusive, one can imagine that the production of extracellular polysaccharides might be hampered during the dispersal stage. In support of this hypothesis, deletion of *hmsHFR*, which is required for the production of β-GlcNAc, leads to enhanced motility (Fig. 6a). To further test this hypothesis, we constructed a promoter-replacement mutant Δ*P*_*hms*_(*P*_*flhDC*_) in which the *hmsHFRS* promoter was replaced by the *flhDC* promoter. As expected, the promoter-replaced mutant that allowed both *flhDC* and *hmsHFRS* to be controlled by the *flhDC* promoter exhibited dramatically reduced motility compared with the wild-type strain (Fig. 6a). In contrast, the biofilm developed on *C. elegans* by the Δ*P*_*hms*_(*P*_*flhDC*_) strain was enhanced compared to the wild-type strain (Fig. 6b). These data suggest that coordinated regulation of *flhDC* and *hmsHFRS* is crucial for the transition between the planktonic and biofilm states.

## Discussion

In this study, we revealed a new motility/biofilm switch, RovM, a LysR family transcriptional regulator that facilitates transition from biofilm to motility via enhancing the expression of *flhDC* and repressing the expression of *hmsHFRS* directly. RovM was recently shown to act as both an activator and repressor in fine-tuning the expression of the T6SS4- and AR3-dependent acid survival system^33^. Herein we demonstrated that RovM repressed the expression of the β-GlcNAc synthesis operon *hmsHFRS* by directly binding to a region overlapping the −35 element in the *hmsHFRS* promoter but activated the expression of the flagella master regulator operon *flhDC* by binding a region 61 bp upstream of the −35 element in the *flhDC* promoter. RovM has been shown to repress RovA expression by recognizing a binding region within the *rovA* promoter, which includes two palindromic sequences (−63 to −53: ATcaTTT-N_5_-AAAgaAT; −62 to −55: TCaTT-N_6_-AAaGA) with similarity to the conserved T-N_11_-A core motif of LysR-type binding sites^39, 40^. Similar palindromic sequences were also identified in the RovM binding sites on the *flhDC* promoter (−186 to −199: TAAgT-N_3_-AaTTA) (Fig. 1e) and the *hmsHFRS* promoter (−38 to −23: ATAT-N_7_-ATAT) (Fig. 3e). Although a number of LysR-type regulators have been shown to act as both activators and repressors in other bacteria^41^, this is the first demonstration that a LysR-type regulator plays a role in inversely regulating the motility- and biofilm formation-related genes, depending on the localization of the binding site on the target promoter.

For pathogenic bacteria, biofilm formation was found to play pivotal roles in the pathogenicity of important pathogens *Staphylococcus epidermidis* ^42, 43^, *Pseudomonas aeruginosa* ^44^ and *Salmonella enterica* serovar*Typhimurium* ^45^. RovM has been shown to repress the RovA-dependent expression of internalization factor invasion^32^. Accordingly, we found that deletion of *rovM* gene leads to hyper-biofilm formation and increase the virulence of *Y. pseudotuberculosis* to both silkworm larvae and mice. Intriguingly, Δ*rovM*Δ*hmsHFR* double mutant failed to form biofilm and became almost completely avirulent in both silkworm larvae and mice infection models. The loss of virulence of Δ*rovM*Δ*hmsHFR* can result from lacking the ability to form biofilm, suggesting that this decreased virulence was directly related to biofilm accumulation, and RovM affects *Y. pseudotuberculosis* virulence is partially dependent on regulation of *hms* genes. Therefore, this finding provided a new perspective for revealing the mechanisms of regulation of bacterial virulence by RovM in pathogenesis.

The expression of *rovM* is very high in minimal medium but strongly inhibited during growth in complex media^24, 32^. Indeed, as a nutrient-sensing regulator, the expression of *rovM* was growth phase-dependent. Expression was observed in the exponential phase of growth, reached a maximum in the post-exponential-phase (Fig. S2a,b). And this nutrient-dependent expression has been shown to be controlled by CsrA. In minimal media, CsrA activates RovM expression, leading to repression of RovA^46^. The CsrA-RovM-RovA regulatory cascade was further shown to be regulated by the cAMP-Crp complex, which links nutrient availability to CsrA activity via activation of CsrC. Deletion of *crp* strongly affects the levels of CsrC and results in the strong upregulation of RovM and repression of RovA^46^. The regulatory activity of CsrA is antagonized by two small noncoding RNAs, CsrB and CsrC, which contain multiple CsrA binding sites that sequester CsrA dimers away from target mRNAs^47–49^. Thus, RovM forms a unique switch that inversely regulates motility and biofilm formation at the transcriptional level by sensing nutrient availability mediated by Crp.

**Figure 7:**
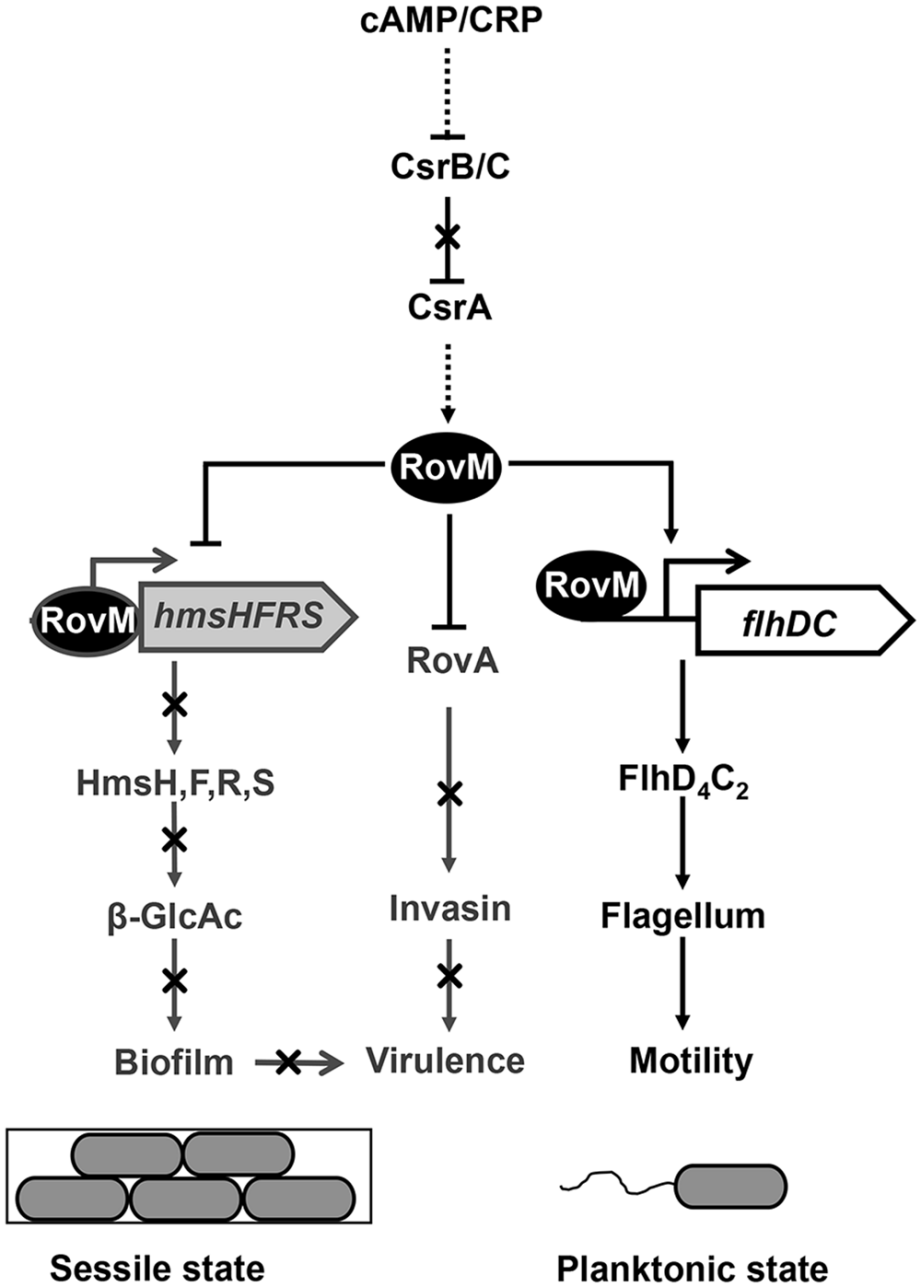
Model for controlling biofilm/motility transition by RovM. CsrA is activated by the cAMP-Crp complex through reducing the amount of CsrB/C. RovM regulator activated by CsrA enhances bacterial motility by directly activating *flhDC* expression while inhibiting β-GlcNAc accumulation by directly repressing the expression of the *hmsHFRS* operon. Thus, RovM formed a switch modulates motility/biofilm transition on transcriptional level. In the meanwhile, RovM represses bacterial virulence through negative regulation of RovA and inhibition of biofilm formation.

Based on our results, we proposed a model in which the RovM acting as a switch controls *Y. pseudotuberculosis* state transition by inversely regulating motility and biofilm formation in response to nutrient status (Fig. 7). As nutrient availability is limited, CsrA is activated by the cAMP-Crp complex through reducing the amount of CsrB/C. RovM regulator activated by CsrA enhances bacterial motility by directly activating *flhDC* expression while inhibiting β-GlcNAc accumulation by directly repressing the expression of the *hmsHFRS* operon. Thus, RovM formed a switch modulates motility/biofilm transition on transcriptional level. In the meanwhile, RovM represses bacterial virulence through negative regulation of RovA and inhibition of biofilm formation as observed in Fig. 7.

However, in *Y. pestis*, cellular RovM level was found to change following a temperature shift from 37 °C (warm-blooded host temperature) and 26 °C (flea gut temperature)^35^, whereas RovM expression was not shown to be temperature-dependent in *Y. pseudotuberculosis* ^32^. The plague bacillus *Y. pestis* is evolved from *Y. pseudotuberculosis* 1,500–6,400 years ago and transmitted by fleas^50, 51^. During being transmitted by flea, *Y. pestis* is not toxic to fleas, whereas *Y. pseudotuberculosis* exhibits significant oral toxicity to the flea vectors of plague^52^. The highly induced expression of *rovM* in *Y. pestis* by sensing temperature signals plays an important signal in abolishing the bacterial toxicity through inhibiting the expression of the major virulence transcriptional regulator *rovA* ^35^. Thus, the development of temperature-dependent *rovM* expression in *Y. pestis* facilitates its adaptation to the flea-borne transmission route.

Since the regulation of biofilm formation by *Y. pestis* is an important characteristic in its flea-borne transmission, developments have to get evolved changes to acquire efficient adhere to flea proventriculus during its evolution from *Y. pseudotuberculosis*. RcsA and NghA that strongly repress biofilm formation in *Y. pseudotuberculosis*, are thought to represent for anti-transmission factors due to loss of function in *Y. pesis* ^53, 54^. The acquisition of pCD1 virulence plasmid during evolution also results in the opposite phenotypes of β-GlcNAc production, even in different *Y. pestis* species^55^. The chaperone RNA-binding protein Hfq was reported to inhibit biofilm development in pCD1 deficient *Y. pestis* strain CO92^56^ while enhances biofilm formation in pCD1-cured *Y. pestis* strain 201^35^. Similarly, RovM was found to activate biofilm accumulation in *Y. pestis* strain 201^35^, whereas the deletion of *rovM* gene in pCD1 deficient *Y. pestis* strain KIM6 + does not affect biofilm formation^57^. Notably, different from the behaviour of RovM from *Y. pestis* strain 201^35^ which positively regulates biofilm formation through regulating *hmsHFRS*, *Y. pseudotuberculosis* YPIII represses biofilm formation through blocking *hmsHFRS* transcription in a directly manner. This difference may be caused by genetic backgrounds and the presence of pCD1 plasmid. Furthermore, the RovM protein from *Y. pestis* strain 201 is found to possess four more Arginine residues in 35^th^ to 39^th^ position comparing with the RovM from YPIII (Fig. S3). The highly conserved “A_6_” motif in YPIII turns into “A_10_” in *Y. pestis* strain 201^58^, and this may also lead to a reversely functional alteration. During the evolution of *Y. pestis*, differential regulation of RovM by environmental signals, as well as functional mutation of RovM on biofilm formation were likely subject to strong Darwinian (positive) selection during the early adaptation of *Y. pestis* to the new transmission route. Altogether, this implies that the development on regulation behave of RovM regulator plays a key role during the evolution of *Y. pestis* into a flea-borne pathogen from *Y. pseudotuberculosis*.

In conclusion, as a distinct motility/biofilm switch, the RovM represses biofilm formation enhances motility directly depending on the localization of the binding site on their promoters. The regulation is subjected to precisely control of bacteria lifecycle in response to nutrient levels, and in turn modulates *Yersinia* pathogenicity more rigorously. During evolution from the progenitor *Y. pseudotuberculosis* to the deadly *Y. pestis*, the evolved change on RovM seems to be one of the key steps that are important for flea-borne transmission of *Y. pestis*. Totally, regulation of RovM allows immediately adaptation of bacteria to ever-changing environments and is crucial for efficiently regulating of bacterial virulence.

## Methods

### Ethics statement

All mouse experimental procedures were performed in accordance with the Regulations for the Administration of Affairs Concerning Experimental Animals approved by the State Council of People’s Republic of China. The protocol was approved by the Animal Welfare and Research Ethics Committee of Northwest A&F University (protocol number: NWAFU 2014002).

### Bacterial strains and growth conditions

Bacterial strains and plasmids used in this study are listed in Supplementary Table S1. *E. coli* strains were cultured in Luria–Bertani (LB) and *Y. pseudotuberculosis* strains were cultured in Yersinia–LB (YLB) broth or M9 medium as previous reported^33^. In-frame deletions were generated by means of the method described by Wang *et al*.^59^.

### Plasmid construction

Primers used in this study are listed in Supplementary Table 2. To construct the *lacZ* fusion reporter vector pDM4-*P*_*hmsHFRS*_ *::lacZ*, primers P*hms*-3F/P*hms*-3R were used to amplify the 503 bp *hmsHFRS* promoter fragment from *Y. pseudotuberculosis* genomic DNA. The PCR product was digested with SalI/XbaI and inserted into similarly digested pDM4-*lacZ* to produce pDM4-*P*_*hmsHFRS*_ *::lacZ*. pDM4-P_*rovM*_::lacZ was constructed in a similar manner using primers P_*rovM*_-F/P_*rovM*_-R.

The Δ*rovM* and Δ*flhDC* in-frame deletion mutant of *Y. pseudotuberculosis* were made in our previous study^33, 38^. The plasmid pDM4-Δ*hmsHFR* (*ypk_2241-2239*) was used to construct the Δ*hmsHFR* in-frame deletion mutant of YPIII. A 761-bp upstream fragment and a 702-bp downstream fragment of *hmsHFR* operon were amplified using the primer pair *hms*-F/*hms*-MR and *hms*-MF/*hms*-R, respectively. The upstream and downstream PCR fragments were ligated by overlapping PCR. The resulting PCR products were digested with SalI and BglII and inserted into the SalI/BglII site of pDM4 to produce pDM4-Δ*hmsHFR*.

To construct the pUC18T-mini-Tn7T-Gm-*rovMvsvG* plasmid, primers *P*_*rovMvsvg*_ *-*F/*P*_*rovMvsvg*_ *-*R were used to amplify the vsvg-tagged *rovM* gene fragment including its native promoter from the YPIII genome. The PCR product was digested with HindIII/BglII and was inserted into HindIII/BamHI pUC18T-mini-Tn7T-Gm. The *Y. pseudotuberculosis* YPIII(*rovM-vsvg*) strain expressing vsvg-tagged RovM was constructed by co-transformation of pTNS3 (50 ng) and pUC18T-mini-Tn7T-Gm-*rovMvsvG* (50 ng) plasmids into the YPIII wild-type strain as described^60^.

To replace the *hmsHFRS* promoter with *flhDC* promoter in *Y. pseudotuberculosis*, the plasmid pDM4-*ΔP*_*hmsHFRS*_(*P*_*flhDC*_) was construct. A 957-bp upstream fragment and a 924-bp downstream fragment flanking *hmsHFRS* promoter were amplified with primer pairs P_*hms*_-1F/P_*hms*_-1MR and P_*hms*_-1MF/P_*hms*_-1R, and the *flhDC* promoter was amplified with primer pairs P_*flhDC*_-1F/P_*flhDC*_-1R. The upstream PCR fragment of *hmsHFRS* promoter and *flhDC* promoter fragment were ligated by overlap PCR to generate fragment PFP_*hms*_-P_*flhDC*_. The fragment PFP_*hms*_-P_*flhDC*_ and the downstream fragment of *hmsHFRS* promoter were ligated by overlap PCR with primer pairs P_*hms*_-1F/P_*hms*_-1R to generate fragment PFP_*hms*_-P_*flhDC*_-PRP_*hms*_. The PFP_*hms*_-P_*flhDC*_-PRP_*hms*_ fragment was digested with SalI/BglII and inserted into the SalI/BglII site of pDM4 to produce pDM4-*ΔP*_*hmsHFRS*_(*P*_*flhDC*_).

To complement the Δ*rovM* mutant, primers *rovM*-F/*rovM*-R were used to amplify the *rovM* gene fragment including its native promoter from the YPIII genome. The PCR product was digested with BamHI/SalI and was inserted into similarly digested pKT100. For complementation and overexpression, plasmids pKT100-*rovM* was introduced into respective strains by electroporation. The integrity of the insert in all constructs was confirmed by DNA sequencing.

### Overexpression and purification of recombinant protein

To express and purify His_6_-RovM, plasmid pET15b-*rovM* was transformed into the *E. coli transB*(DE3) competent cells. For protein production, bacteria were grown at 37 °C in LB medium to an OD_600_ of 0.5. The strains were then induced with 0.2–0.4 mM isopropyl β-D-1-thiogalactopyranoside (IPTG) and cultivated for an additional 16 h at 22 °C. Harvested cells were disrupted by sonication and purified with the His**·**Bind Ni-NTA resin (Novagen, Madison, WI, USA), according to the manufacturer’s instructions. Purified recombinant proteins were dialyzed in phosphate-buffered saline (PBS) overnight at 4 °C and stored at −80 °C until use. The purity of the purified protein was verified as >95% homogeneity based on SDS-PAGE analysis. Protein concentrations were determined using the Bradford assay^61^.

### Construction of chromosomal fusion reporter strains and β-galactosidase assays

The *lacZ* fusion reporter vectors pDM4-*P*_*hmsHFRS*_ *::lacZ*, pDM4-*P*_*flhDC*_ *::lacZ* and pDM4-*P*_*rovM*_ *::lacZ* were transformed into *E. coli* S17–1λpir and mated with *Y. pseudotuberculosis* strains according to the procedure described previously^59^. The *lacZ* fusion reporter strains were grown in YLB or M9 broth and β-galactosidase activities were assayed with *o*-nitrophenyl-β-galactoside (ONPG) as substrate. The assays were performed in triplicate at least three times, and error bars represent standard deviation. Statistical analysis was carried out with Student’s *t*-test.

### Biofilm assay

Biofilm formation of *Y. pseudotuberculosis* strains (labelled with plasmid pKEN-GFP mutant3*) on *C. elegans* was assayed as described^62^. Biofilm accumulation was classed as level 0-3. The level of biofilm accumulation on *C. elegans* was denoted as the biofilm severity incidence and was calculated as previously described^63^: Biofilm severity incidence = {[∑(level X number of samples in this level)]/(highest level X total sample numbers)} × 100%. Biofilm formation on abiotic surface was assayed in 96-well polystyrene microtiter plates as previously described^64^.

### Congo red assay, EPS quantification and WGA-R staining assay

Two OD of an overnight culture grown at 26 °C in M9 medium was collected and washed using ddH_2_O 3 times. Subsequently, sediments were suspended with 0.4% congo red solution and incubated for 30 min at 37 °C. Nonspecifically bound congo red was removed by washing with 1 M NaCl for 20 min, and the bacteria were washed with ddH_2_O three times. Finally, the bacteria were suspended in 1 ml ddH_2_O. Extracellular polysaccharide was quantified by 3-methyl-2-benzothiazolone hydrazone hydrochloride (MBTH) method as previously described^65^. The presence of the β-GlcNAc in the extracellular of *Y. pseudotuberculosis* aggregates was demonstrated using a WGA-R conjugate as previously reported^27^.

### Electron microscopy

For field emission scanning electron microscopy, glass coverslips were coated with a poly-L-lysine solution, and the samples were fixed in 2% glutaraldehyde in cacodylate buffer. Dehydration was performed in a graded series of acetone concentrations (10%, 30%, 50%, 70%, 90%, 100%) on ice for 15 min for each step. Samples were then critical point dried with liquid CO_2_ and covered with a gold film by sputter coating. Examination was performed with a field emission scanning electron microscope (Hitachi S4800).

### Electrophoretic mobility shift assay (EMSA) and DNase I footprinting assay

EMSA was performed as described previously using biotin 5′-end labelled promoter probes^66^. Fragments Bio-P_*flhDC*_, Bio-P_*hmsHFRS*_, P_*flhDC*_, P_*hmsHFRS*_ and their unrelated fragments (URDs) were amplified from the genomic DNA of YPIII, with primers *flhDC*-biotinF/*flhDC*-biotinR, *hms*-biotinF/*hms*-biotinR, *flhDC*-emsaF/*flhDC*-emsaR, *hms*-emsaF/*hms*-emsaR, *flhDC*-URD F/*flhDC*-URD R, and *hms*-URD F/*hms*-URD R, respectively. All PCR fragments were purified by EasyPure Quick Gel Extraction Kit (TransGen Biotech, Beijing, China). Each 20-μl EMSA reaction solutions were prepared by adding the following components according to the manufacturer’s protocol (Light Shift Chemiluminescent EMSA kit; Thermo Fisher Scientific). 20 fmol Biotin-DNA, 4 pmol unlabelled DNA as competitor and different concentrations of proteins [0, 0.13, 0.27, 0.54 and 0.108 µM]. Reaction solutions were incubated for 20 min at room temperature. The protein-probe mixture was separated in a 6% polyacrylamide native gel and transferred to a Biodyne B Nylon membrane (Thermo Fisher Scientific). Migration of biotin-labelled probes was detected by streptavidin-horseradish peroxidase conjugates that bind to biotin and chemiluminescent substrate according to the manufacturer’s protocol. DNase I footprinting assays were performed according to Wang *et al*.^59^.

### Quantitative Real-Time PCR (qRT-PCR)

qRT-PCR analysis was performed as described previously^33^.

### Western blot analysis

Western blot analysis was performed as described previously^38^. Samples were resolved by SDS-PAGE and transferred onto polyvinylidene fluoride membranes (Millipore). The membrane was blocked in 5% (w/v) non-fat milk for 4 h at room temperature and incubated with primary antibodies at 4 °C overnight: anti-VSVG (Santa Cruz Biotechnology), 1:500; anti-RNA pol β (Santa Cruz Biotechnology). The membrane was washed three times in TBST buffer (50 mM Tris, 150 mM NaCl, 0.05% Tween 20, pH 7.4) and incubated with a 1:5000 dilution of horseradish peroxidase-conjugated secondary antibodies (Shanghai Genomics) for 1 h. Signals were detected using the ECL plus kit (GE Healthcare) following the manufacturer’s protocol.

### Mouse infections

All mice were maintained and handled in accordance with the animal welfare assurance policy issued by Northwest A&F University. Post-exponential phase *Y. pseudotuberculosis* strains grown in M9 medium at 26 °C, washed twice in sterilized PBS and used for orogastric infection of 6–8 weeks old female C57BL/6 mice using a ball-tipped feeding needle. For survival assays 3 × 10^9^ bacteria of each strain were applied to different groups of mice (10/group) for one time, and the survival rate of the mice was determined by monitoring the survival everyday for 21 days^59^.

### Silkworm rearing and infection

The silkworm *Bombyx mori* (Nistari strain) was reared on mulberry leaves at 27 °C in 70% RH and a photo period of 13:11 (light:dark). *Y. pseudotuberculosis* strains were grown to post-exponential phase in M9 medium at 26 °C. The cells were collected by centrifugation at 8000× *g*. The pellet was washed twice in sterilized PBS and 5 × 10^8^ bacteria were injected into each hemocoel of day-3 fifth-instar silkworm for one time. Each group contains 25 silkworms. And the survival rate of the silkworm was determined by monitoring the survival every 12 hours for 6 days^67^.

### Statistical analysis

Statistical analyses were performed using paired two-tailed Student’s *t*-test. Survival times were analyzed using Kaplan-Meyer curves and comparisons were performed using the Log-Rank test. Statistical analyses were performed using GraphPad Prism Software (GraphPad Software, San Diego California USA).

## Electronic supplementary material

Supplementary information
