# Supplementary material for: A starvation-induced regulator, RovM, acts as a switch for planktonic/biofilm state transition in Yersinia pseudotuberculosis
Source: Sci Rep. 2017 Apr 4;7:639. doi: 10.1038/s41598-017-00534-9 (PMC5428675; doi:10.1038/s41598-017-00534-9)
Supplement: Supplementary file 1 — Supplementary information [file 41598_2017_534_MOESM1_ESM.pdf]

Supplementary Information

A starvation-induced regulator, RovM, acts as a switch for planktonic/biofilm state transition in *Yersinia pseudotuberculosis*

Ruoxi Zhao[1#], Yunhong Song[1#], Qingyun Dai[1], Yiwen Kang[1], Junfeng Pan[1], Lingfang Zhu[1], Lei Zhang[1], Yao Wang[1]*, XihuiShen[1]*

[1]State Key Laboratory of Crop Stress Biology for Arid Areas and College of Life Sciences, Northwest A&F University, Yangling, Shaanxi 712100, China

Running title: RovM regulates planktonic/biofilm state transition

[#] These authors contributed equally to this work.

*Corresponding authors

xihuishen@nwsuaf.edu.cn and wangyao@nwsuaf.edu.cn

Key words: *Yersinia pseudotuberculosis*, RovM, biofilm, motility, pathogenicity, nutrient

Supplementary Table 1. Bacterial strains and plasmids used in this study.

Strains	Relevant characteristics	Reference
E. coli		
S17-1*λ pir*	*λ*-pir lysogen of S17-1, *thi pro hsdR hsdM*$^+$ *recA* RP4 2-Tc::Mu-Km::Tn7	Simon *et al.*, 1983
JM109	*recA1 supE44 endA1 hsdR17 gyrA96 relA1 thi*Δ(*lac-proAB*)F'(*traD36 proABlacI*^{q}*lacΔZM15*)	Stratagene
OP50	Uracil auxotroph which serves as a nutrient source for *C. elegans*	Lewis and Fleming, 1995
*Trans*B(DE3)	Host for expression vector pET15b	TransGen Biotech
Y. pseudotuberculosis		
YPIII	Wild-type *Y. pseudotuberculosis* pIB1, Nal^r	Rosqvist *et al.*, 1988
Δ*rovM*	*rovM* gene deleted in YPIII, Nal^r	Song *et al.*, 2015
Δ*flhDC*	*flhDC* gene deleted in YPIII, Nal^r	Xu *et al.*, 2014
Δ*hmsHFR*	*hmsHFR* gene deleted in YPIII, Nal^r	This study
Δ*rovM*Δ*flhDC*	*rovM*, *flhDC* double genes deleted in YPIII, Nal^r	This study
Δ*rovM*Δ*hmsHFR*	*rovM*, *hmsHFR* double genes deleted in YPIII, Nal^r	This study
Δ$P_{hmsHFRS}$(P_{flhDC})	Replace *hmsHFRS* promoter with *flhDC* promoter in YPIII,	This study
YPIII(vector)	YPIII containing pKT100, Nal^r, Km^r	This study
YPIII(*rovM-vsvg*)	YPIII expressing vsvg-tagged RovM on chromosome	This study
Δ*rovM*(vector)	Δ*rovM* containing pKT100, Nal^r, Km^r	This study
Δ*flhDC*(vector)	Δ*flhDC* containing pKT100, Nal^r, Km^r	This study
Δ*hmsHFR*(vector)	Δ*hmsHFRS* containing pKT100, Nal^r, Km^r	This study
Δ*rovM*(*rovM*)	Δ*rovM* containing pKT100-*rovM*, Nal^r, Km^r	This study
Δ*rovM*Δ*flhDC*(*rovM*)	Δ*rovM*Δ*flhDC* containing pKT100-*rovM*, Nal^r, Km^r	This study
Δ*rovM*Δ*hmsHFR*(*rovM*)	Δ*rovM*Δ*hmsHFR* containing pKT100-*rovM*, Nal^r, Km^r	This study
**Plasmid**		
pKT100	Cloning vector, p15A replicon, Km^r	Hu *et al.*, 2009
pKEN-*gfp* mut3*	pH-sensitive green fluorescent protein mutant3* expressed in pKEN, Amp^r	Wilks*et al.*, 2007
pKT100-*rovM*	*rovM* under the control of its native promoter in plasmid pKT100, Km^r	Song *et al.*, 2015
pET15b	Expression vector with N-terminal hexahistidine affinity tag, Amp^r	Novagen
pET15b-*rovM*	pET15b carrying *rovM* coding region, Amp^r	Song *et al.*, 2015
pUC18T-mini-Tn7T-Gm	Gm^r on mini-Tn7T; mobilizable; for gene insertion in Gms bacteria	Kim *et al.*, 2006
pUC18T-mini-Tn7T-Gm-*rovMvsvG*	*rovM* fusion with *vsvG* tag under the control of self promoter in plasmid pUC18T-mini-Tn7T-Gm	This study
pTNS3	Helper plasmid DNA for pUC18T-mini-Tn7T-Gm	Kim *et al.*, 2006
pDM4	Suicide vector, *mob*RK2, *ori*R6K, *pir*, *sacB*, Cm^r	Milton *et al.*, 1996
pDM4-Δ*hms*	Construct used for in-frame deletion of *hmsHFR*, Cm^r	This study
pDM4-Δ*flhDC*	Construct used for in-frame deletion of *flhDC*, Cm^r	Xu *et al.*, 2014
pDM4-Δ$P_{hmsHFRS}$(P_{flhDC})	Consturct used for replacing *hmsHFRS* promoter with *flhDC* promoter, Cm^r	This study
pDM4-$P_{hmsHFRS}$::*lacZ*	*hmsHFRS::lacZ* fusion in pDM4	This study
pDM4-P_{flhDC}::*lacZ*	*flhDC::lacZ* fusion in pDM4	Ding *et al*. 2009
pDM4-P_{rovM}::*lacZ*	*rovM::lacZ* fusion in pDM4	This study

*Nal^r, Cm^r, Km^r, and Amp^r represent resistance to naladixic acid, chloramphenicol, kanamycin, gentamycin and ampicillin at 20, 20, 50, 15 and 100 μg ml^{-1}, respectively.

Supplementary Table 2. Primers used in this study.

Primers	5'-3' sequence	
hms-F	GAGTCGACGGGGAAATTTCCAATCAG	To generate pDM4-Δ*hms*
hms-MR	**GATGACGGCACAGGCTGG**GGCGGAACGAACGTTGGC	To generate pDM4-Δ*hms*
hms-MF	**GCCAACGTTCGTTCCGCC**CCAGCCTGTGCCGTCATC	To generate pDM4-Δ*hms*
hms-R	ACGCAGATCTCGGTACCCAATGCATGGC	To generate pDM4-Δ*hms*
P_{hms}-1F	ACGCGTCGACGTACTGAAAACGTTTCCGG	To generate pDM4-Δ$P_{hmsHFRS}$(P_{flhDC})
P_{hms}-1MR	TTAGAAATACCGACGTAAGTCACGA	To generate pDM4-Δ$P_{hmsHFRS}$(P_{flhDC})
P_{flhDC}-1F	**TCGTGACTTACGTCGGTATTTCTAA**GGGGGCGCGGTTTCAAATAG	To generate pDM4-Δ$P_{hmsHFRS}$(P_{flhDC})
P_{flhDC}-1R	**GTTGTAAATGCGTTATACAT**CTTACACATCCCAACTGATT	To generate pDM4-Δ$P_{hmsHFRS}$(P_{flhDC})
P_{hms}-2MF	ATGTATAACGCATTTACAAC	To generate pDM4-Δ$P_{hmsHFRS}$(P_{flhDC})
P_{hms}-2R	GGAAGATCTCGTCTGATGACGGACCAATA	To generate pDM4-Δ$P_{hmsHFRS}$(P_{flhDC})
P_{hms}-3F	ACGCGTCGACATTAAAATTGTAATGATT	pDM4-$P_{hmsHFRS}$::*lacZ*
P_{hms}-3R	GCTCTAGACATTATATAACCCTTAAG	pDM4-$P_{hmsHFRS}$::*lacZ*
P_{rovM}-F	GTGCGTCGACATGTGGGCTAGATCCATCCG	To generate pDM4-*ProvM::lacZ*
P_{rovM}-R	GTGCTCTAGAGTCGAGATTAATTATCGGACGA	To generate pDM4-*ProvM::lacZ*
$P_{rovMvsvg}$-F	GGGGCCATGTGGGCTAGATCCATCCG	To generate pUC18T-mini-Tn7T-Gm-*rovMvsvG*
$P_{rovMvsvg}$-R	GAAGATCTTCATTTTCCTAATCTATTCATTTCAATATCTGTATAATCTTCATCACCTGTC	To generate pUC18T-mini-Tn7T-Gm-*rovMvsvG*
hms-biotinF	GTCCCTTGAAATCCGTTC (5'-biotin modified)	For EMSA
hms-biotinR	TATATAACCCTTAAGCCAGCA (5'-biotin modified)	For EMSA
hms-emsaF	GTCCCTTGAAATCCGTTC	For EMSA
hms-emsaR	TATATAACCCTTAAGCCAGCA	For EMSA
hms-URD F	GCGCTATTTATTTCTGGCTT	For EMSA
hms-URD R	CGCTGATAAGATCAGGAT	For EMSA
flhDC-biotinF	GCTCTGATATTTCAGTTTGGG (5'-biotin modified)	For EMSA
flhDC-biotinR	CTTACACATCCCAACTGATTATG (5'-biotin modified)	For EMSA
flhDC-emsaF	GCTCTGATATTTCAGTTTGGG	For EMSA
flhDC-emsaR	CTTACACATCCCAACTGATTATG	For EMSA
flhDC-URD F	CGTCTGAATTACTCAAAC	For EMSA
flhDC-URD R	TAAAGCCGATACGCTCTA	For EMSA
flhD-F	TTTGTCACTTCCGTTTT	qRT-PCR
flhD-R	ATGCTCTTTTTTTAGGC	qRT-PCR
flhC-F	CCGATTGGTTTATGACTTG	qRT-PCR
flhC-R	CGGGACTTCAGGCAGAT	qRT-PCR
hmsH-F	TTTGCTTGATGCCGATGA	qRT-PCR
hmsH-R	ACCTGATACGGCGTTTGC	qRT-PCR
hmsS-F	ATCAGGTCCGTTTCCAGG	qRT-PCR
hmsS-R	TGAGACACGAGCCACTTT	qRT-PCR
16S RNA-F	CTAGCGATTCCGACTTCAT	qRT-PCR
16S RNA-R	CCCTTATCCTTTGTTGCC	qRT-PCR

Underlined sites indicate restriction enzyme cutting sites added for cloning. Letters in boldface denote the annealing regions for overlap PCR.

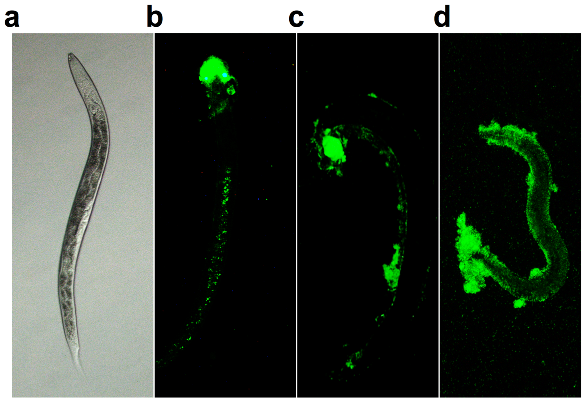

Figure S1. Biofilm formation of *Y. pseudotuberculosis* strains on the surface of *C. elegans.* *C. elegans* were infected with GFP-labelled post-exponential bacteria, and biofilms formed on the worms were detected under low magnification using a fluorescent microscope at 24 h post-infection. (**a-d**). Four representative images of increasingly severe (0, 1, 2, 3) biofilm formation on *C. elegans*. Level 0 represents no biofilm formation on *C. elegans*; level 1 indicates a small amount of biofilm formation around the worms' anterior; level 2 indicates larger accumulation of biofilm around the anterior end and some spreading back from the head; and level 3 indicates that biofilm extend to parts of the body other than the head.

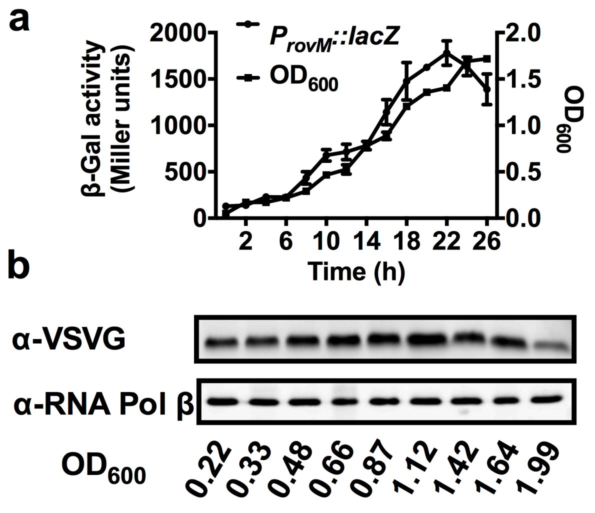

Figure S2. RovM is starvation induced. (**a**) β-Galactosidase activity of *rovM* promoter was detected at different growth stages in M9 medium. (**b**) The expression of C-terminal VSV-G-tagged RovM was detected by western blot at different growth stages in M9 medium. The loading control detected by anti-RNA pol β was shown for total protein lysate. Data shown are the average of three independent experiments; error bars indicate SD from three independent experiments. **, $P<0.01$.

Score	Expect	Method	Identities	Positives	Gaps
633 bits(1632)	0.0()	Compositional matrix adjust.	310/314(99%)	310/314(98%)	4/314(1%)

```
YpIII           1    MTNANRPIINLDLDLLRTFVAVADLNTFAAAAAA----VCRTQSAVSQQMQRLEQLVGKE   56
                     MTNANRPIINLDLDLLRTFVAVADLNTFAAAAAA    VCRTQSAVSQQMQRLEQLVGKE
Y. pestis 201   1    MTNANRPIINLDLDLLRTFVAVADLNTFAAAAAAAAAAVCRTQSAVSQQMQRLEQLVGKE   60

YpIII           57   LFARHGRNKLLTEHGLQLLGYARKILRFNDEACTSLMYSNMEGSLIIGASDDTADTLLPF   116
                     LFARHGRNKLLTEHGLQLLGYARKILRFNDEACTSLMYSNMEGSLIIGASDDTADTLLPF
Y. pestis 201   61   LFARHGRNKLLTEHGLQLLGYARKILRFNDEACTSLMYSNMEGSLIIGASDDTADTLLPF   120

YpIII           117  LLNRVATLYPRLAIDVRVKRSPFIADMLSSGEVDLAITTAKVDSHPHVILRTSPTLWYCS   176
                     LLNRVATLYPRLAIDVRVKRSPFIADMLSSGEVDLAITTAKVDSHPHVILRTSPTLWYCS
Y. pestis 201   121  LLNRVATLYPRLAIDVRVKRSPFIADMLSSGEVDLAITTAKVDSHPHVILRTSPTLWYCS   180

YpIII           177  VDYQFQPGEPVPLVVMDEPSLYREMAIEHLTQAGVPWRIAYVASSLSAIRAAVRAGLGVT   236
                     VDYQFQPGEPVPLVVMDEPSLYREMAIEHLTQAGVPWRIAYVASSLSAIRAAVRAGLGVT
Y. pestis 201   181  VDYQFQPGEPVPLVVMDEPSLYREMAIEHLTQAGVPWRIAYVASSLSAIRAAVRAGLGVT   240

YpIII           237  ARPIEMMSPDLRVLGETEGLPGLPETRYVLCKDKQCDNELALAIFSALQNSYQHTMSSES   296
                     ARPIEMMSPDLRVLGETEGLPGLPETRYVLCKDKQCDNELALAIFSALQNSYQHTMSSES
Y. pestis 201   241  ARPIEMMSPDLRVLGETEGLPGLPETRYVLCKDKQCDNELALAIFSALQNSYQHTMSSES   300

YpIII           297  SLILDSDYLTGDED   310
                     SLILDSDYLTGDED
Y. pestis 201   301  SLILDSDYLTGDED   314
```

Figure S3. Comparison of the amino acid sequence of RovM from *Y. pseudotuberculosis* YpIII and *Y. pestis* 201. The RovM protein from *Y. pestis* strain 201 possesses four more Arginine residues in position 35th to 39th compared to the RovM from *Y. pseudotuberculosis* YpIII. The different sequence was indicated by red box. The highly conserved "A_6" motif in YPIII turns into "A_{10}" in *Y. pestis* strain 201.

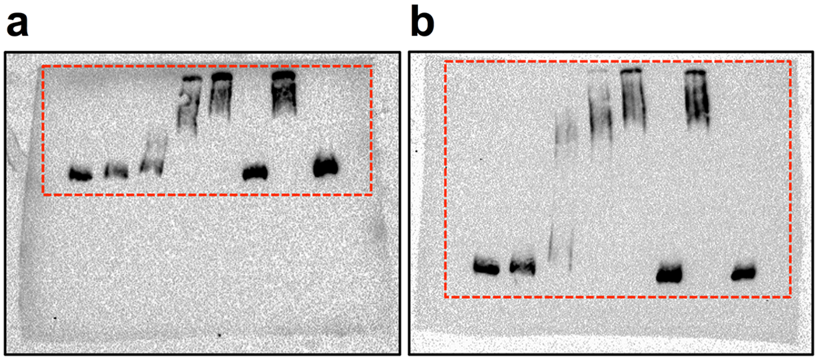

Figure S4. Uncropped versions of scans of Figure 1c (a) and Figure 3c (b).

Supplementary References:

1. Ding, L.S. *et al*. Functional characterization of FlgM in the regulation of flagellar synthesis and motility in *Yersinia pseudotuberculosis*. *Microbiology* **155,** 1890-1900(2009).

2. Hu, Y., Lu, P., Wang, Y., Ding, L., Atkinson, S.& Chen, S. OmpR positively regulates urease expression to enhance acid survival of *Yersinia pseudotuberculosis. Microbiology* **155,** 2522-2531(2009).

3. Kim J S, Choi S H, Lee J K. Lysine decarboxylase expression by *Vibrio vulnificus* is induced by SoxR in response to superoxide stress. *J Bacteriol* **188,** 8586-8592 (2006).

4. Lewis, J. A. &Fleming, J. T. *Caenorhabditis elegans*: Modern Biological Analysis of an Organism. New York: Academic Press. p 39 (1995).

5. Milton, D. L., O'Toole, R., Hörstedt, P. & Wolf-Watz, H.Flagellin A is essential for the virulence of *Vibrio anguillarum*. *J. Bacteriol* **178,** 1310–1319(1996).

6. Rosqvist, R., Skurnik, M. & Wolf-Watz, H. Increased virulence of *Yersinia pseudotuberculosis* by two independent mutations. *Nature* **334,** 522–524(1988).

7. Simon, R., Priefer, U. & Puhler, A.A broad host range mobilization system for in vivo genetic engineering: transposon mutagenesis in gram negative bacteria. *Nat Biotechnol* **1,** 784–791(1983).

8. Song, Y. *et al*. The dual transcriptional regulator RovM regulates the expression of AR3- and T6SS4-dependent acid survival systems in response to nutritional status in *Yersinia pseudotuberculosis*. *Environ Microbiol* **17,** 4631-4645(2015).

9. Wilks, J.C. & Slonczewski, J.L. pH of the cytoplasm and periplasm of *Escherichia coli*: rapid measurement by green fluorescent protein fluorimetry. *J Bacteriol* **189,** 5601-5607(2007).

10. Xu, S. *et al*. FliS modulates FlgM activity by acting as a non-canonical chaperone to control late flagellar gene expression, motility and biofilm formation in *Yersinia pseudotuberculosis*. *Environ Microbiol* **16,**

1090-1104(2014).
